# *Bacillus amyloliquefaciens* Regulates the *Keap1/Nrf2* Signaling Pathway to Improve the Intestinal (Caco-2 Cells and Chicken Jejunum) Oxidative Stress Response Induced by Lipopolysaccharide (LPS)

**DOI:** 10.3390/antiox13121550

**Published:** 2024-12-17

**Authors:** Xing Chen, Aijuan Zheng, Shuzhen Li, Zedong Wang, Zhimin Chen, Jiang Chen, Zhiheng Zou, Haijun Liang, Guohua Liu

**Affiliations:** 1 Key Laboratory for Feed Biotechnology of the Ministry of Agriculture and Rural Affairs, Institute of Feed Research, Chinese Academy of Agriculture Sciences, Beijing 100081, China; 82101231286@caas.cn (X.C.); zhengaijuan@caas.cn (A.Z.); 82101211644@caas.cn (S.L.); wangzedong@caas.cn (Z.W.); chenzhimin@caas.cn (Z.C.); 2 Jiangxi Province Key Laboratory of Animal Green and Healthy Breeding, Institute of Animal Husbandry and Veterinary Science, Jiangxi Academy of Agricultural Sciences, Nanchang 330200, China; jiangchen@jxaas.cn (J.C.); zouzh@jxaas.cn (Z.Z.); 3 China Feed Industry Association, Beijing 100193, China

**Keywords:** *Bacillus amyloliquefaciens*, Caco-2 cell, oxidative stress, *Keap1-Nrf2* signaling pathway, antioxidant capacity

## Abstract

This article aims to investigate the mechanism by which *Bacillus amyloliquefaciens* alleviates lipopolysaccharide (LPS)-induced intestinal oxidative stress. The study involved two experimental subjects: human colorectal adenocarcinoma (Caco-2) cells and Arbor Acres broiler chickens. The experiment involving two samples was designed with the same treatment groups, specifically the control (CK) group, lipopolysaccharide (LPS) group, *Bacillus amyloliquefaciens* (JF) group, and JF+LPS group. In the Caco-2 experiment, we administered 2 μg/mL of LPS and 1 × 10^6^ CFU/mL of JF to the LPS and JF groups, respectively. In the broiler experiment, the LPS group (19–21 d) received an abdominal injection of 0.5 mg/kg BW of LPS, whereas the JF group was fed 1 × 10^7^ CFU/g of JF throughout the entire duration of the experiment (1–21 d). The results indicated the following: (1) JF significantly decreased the DPPH free radical clearance rate and hydrogen peroxide levels (*p* < 0.05). (2) JF significantly enhanced the total antioxidant capacity (T-AOC), superoxide dismutase (SOD), and glutathione peroxidase (GSH Px) activity in Caco-2 cells (*p* < 0.05), while concurrently reducing malondialdehyde (MDA) content (*p* < 0.05). (3) Compared to the CK group, JF significantly increased the mRNA expression levels of *nuclear factor-erythroid 2-related factor 2* (*Nrf2*), *heme oxygenase-1* (*HO-1*), SOD, *catalase* (*CAT*), GSH-Px, *interleukin-4* (*IL-4*), *interleukin-10* (*IL-10*), Claudin, Occludin1, *zonula occludens-1* (*ZO-1*), and *mucin 2* (*MUC2*) in Caco-2 cells (*p* < 0.05), while concurrently reducing the mRNA expression of *Kelch-like ECH-associated protein 1* (*Keap1*), *tumor necrosis factor-alpha* (*TNF-α*), *interleukin-1β* (*IL-1β*), and *interleukin-8* (*IL-8*) (*p* < 0.05). In comparison to the LPS group, the JF+LPS group demonstrated a significant increase in the mRNA expression of *Nrf2*, *SOD*, *GSH-Px*, and *IL-4*, as well as *Occludin1*, *ZO-1*, and *MUC2* in Caco-2 cells (*p* < 0.05), alongside a decrease in the mRNA expression of *Keap1*, *TNF-α*, and *IL-1β* (*p* < 0.05). (4) In broiler chickens, the JF group significantly elevated the levels of T-AOC, CAT, and GSH-Px in the jejunum while reducing MDA content (*p* < 0.05). Furthermore, the CAT level in the JF+LPS group was significantly higher than that observed in the LPS group, and the levels of MDA, TNF-α, and IL-1β were significantly decreased (*p* < 0.05). (5) In comparison to the CK group, the JF group exhibited a significant increase in *Nrf2* levels in the jejunum of broiler chickens (*p* < 0.05). Notably, the mRNA expression levels of *IL-4*, *IL-10*, *Claudin*, *Occludin1*, *ZO-1*, and *MUC2* were reduced (*p* < 0.05), while the mRNA expression levels of *Keap1, TNF-α*, and *IL-1β* also showed a decrease (*p* < 0.05). Furthermore, the mRNA expression levels of *Nrf2*, *Occludin1*, *ZO-1*, and *MUC2* in the JF+LPS group were significantly elevated compared to those in the LPS group (*p* < 0.05), whereas the mRNA expression levels of *Keap1* and *TNF-α* were significantly diminished (*p* < 0.05). In summary, JF can enhance the intestinal oxidative stress response, improve antioxidant capacity and intestinal barrier function, and decrease the expression of inflammatory factors by regulating the *Keap1*/*Nrf2* signaling pathway.

## 1. Introduction

Since the 1950s, antibiotics have been extensively utilized in livestock production to enhance the average daily weight gain and feed efficiency [1]. However, the long-term application of antibiotics has led to several adverse effects, including increased antibiotic resistance, the presence of antibiotic residues in animal products, and environmental pollution [2,3]. These issues ultimately have implications for human health. Consequently, various regions worldwide have begun to impose restrictions or bans on the addition of antibiotics to animal feed [4]. In July 2020, China implemented a comprehensive ‘feed ban on antibiotic resistance’. This ban has fostered the development of animal husbandry towards a healthier, more environmentally friendly, and sustainable approach [5]. It encourages livestock farmers to explore and adopt new, safer feed additives, such as bacterial preparations, which can enhance intestinal health, boost animal immunity, and reduce the reliance on antibiotics [6,7].

*Bacillus amyloliquefaciens* is a significant microbial resource within the genus Bacillus [8]. This bacterium is capable of producing a range of metabolites that inhibit fungal and bacterial activity during its growth process, rendering it widely applicable in various fields, including agriculture, animal husbandry, and aquaculture [9]. *Bacillus amyloliquefaciens* significantly enhances the growth performance of broiler chickens, reduces overall mortality, and minimizes intestinal mucosal lesions [10]. Furthermore, Li et al. demonstrated that *Bacillus amyloliquefaciens* not only improves the growth performance of chickens but also significantly decreases the expression of genes encoding inflammatory cytokines [11]. Similarly, supplementation with *Bacillus amyloliquefaciens* enhanced the growth performance and bone health of broiler chickens, akin to the effects of antibiotic growth promoters (AGPs). This improvement is attributed to the upregulation of intestinal phosphate transporters, the modulation of microbiota, and increased phosphorus retention [12].

Currently, numerous research reports focus on the enhancement of broiler growth performance through the use of *Bacillus amyloliquefaciens*. However, the mechanisms by which this bacterium mitigates lipopolysaccharide (LPS)-induced intestinal oxidative stress have yet to be thoroughly investigated. Therefore, this experiment aims to elucidate the mechanisms by which *Bacillus amyloliquefaciens* JF alleviates LPS-induced oxidative stress in human colorectal adenocarcinoma (Caco-2) cells and the jejunum of broilers, thereby providing a theoretical foundation for its application in livestock and poultry.

## 2. Materials and Methods

### 2.1. Cell Experiment

#### 2.1.1. Reagents and Cell Lines

Strain JF was isolated from the rumen fluid of a fistulated cow. The Caco-2 cell line was obtained from Sigma-Aldrich, St. Louis, MO, USA. Dulbecco’s modified Eagle’s medium nutrient mix F12 (DEME/F12), antibiotics (containing 100 U/mL penicillin and 100 U/mL streptomycin), fetal bovine serum (FBS), trypsin-EDTA (0.25%), and phosphate-buffered saline (pH = 7.4) were purchased from Thermo Fisher Scientific Inc. (Grand Island, NY, USA). The complete culture medium was supplemented with 10% FBS and 1% antibiotics based on DMEM/F12. Lipopolysaccharide (LPS) was derived from Escherichia coli 0111: B4 (Sigma-Aldrich, St. Louis, MO, USA).

#### 2.1.2. Bacterial Strain Cultures

The JF strain cryopreservation tube, which contained 15% glycerol, was removed from the −80 °C freezer. The JF strain was then added to 100 mL of sterilized Luria–Bertani (LB) broth (Solarbio, Beijing, China) at a 1% inoculation dose. Following incubation at 37 °C for 24 h, strain JF was purified on LB solid medium for subsequent use.

#### 2.1.3. Bacterial Strain Antioxidant Activity

The antioxidant activity of strain JF was evaluated by its DPPH free radical scavenging ability and hydrogen peroxide (H_2_O_2_) content. The DPPH radical scavenging assay kit (A153-1-1, Spectrophotometer method) and hydrogen peroxide assay kit (A064-1-1, Spectrophotometer method) were obtained from the Nanjing Jiancheng Biotechnology Research Institute Co., Ltd. (Nanjing, China). The activated strain JF was cultured for 18 h until it reached a concentration of 1 × 10^9^ CFU/mL. Subsequently, the JF strain was gradually diluted to a concentration of 10^9^–10^1^ CFU/mL using LB medium. The DPPH radical scavenging ability and hydrogen peroxide content were determined following the reagent supplier’s instructions.

#### 2.1.4. Cell Culture

Caco-2 cell cryovials were removed from liquid nitrogen and melted in a 37 °C water bath. The method is as follows: Once the cells have melted, remove them from the water bath and sterilize the outside of the freezing tube by wiping it with an alcohol cotton ball. Then, place the tube on an ice bath. Open the bottle cap carefully and transfer the cells into a culture dish containing 10 mL of medium. Next, transfer the dish to a culture incubator set at 37 °C with 5% CO_2_. Once the cells adhere to the cell wall and reach approximately 80% confluence, they are digested with trypsin and subsequently passaged.

#### 2.1.5. Cell Treatment

Cultivated Caco-2 cells were seeded into a 6-well plate and homogenized by cross-shaking, and each well was supplemented with 3 mL complete culture medium, containing a cell suspension of 1 × 10^6^/mL. The cells were then cultured for 48 h in an incubator set at 37 °C with 5% CO_2_. Afterward, the culture medium was discarded, and the wells were rinsed three times with PBS. The experiment consisted of four treatment groups: the control (CK group), the *B. amyloliquefaciens* group (JF group), the LPS group (LPS group), and the *B. amyloliquefaciens* + LPS group (JF+LPS group). Each process had 3 replicates. The treatment method for the control group involved adding 3 mL of complete culture medium. In the *B. amyloliquefaciens* group, 3 mL (1 × 10^6^ CFU/mL) *B. amyloliquefaciens* was added, while in the LPS group, 3 mL LPS (2 μg/mL) was added. These 3 groups were then cultivated in a 37 °C with 5% CO_2_ incubator for 12 h. The JF+LPS group treatment method involved adding 2 mL of complete culture medium, which included 1 × 10^6^ CFU/mL *B. amyloliquefaciens*. The mixture was then cultivated in a 37 °C with 5% CO_2_ incubator for 4 h. After discarding the culture medium, the well was washed three times with PBS. Subsequently, 2 mL (2 μg/mL, diluted by complete culture medium solution) LPS was added, and the cultivation was continued for another 12 h.

#### 2.1.6. Cellular Antioxidant Capacity

The CK group, LPS group, JF group, and JF+LPS group were assessed for total antioxidant capacity (T-AOC), malondialdehyde (MDA) levels, and the activities of superoxide dismutase (SOD) and glutathione peroxidase (GSH-Px). The antioxidant test kits used in this study were procured from Nanjing Jiancheng Biotechnology Research Institute Co., Ltd. (Nanjing, China).

#### 2.1.7. Quantitative Real-Time PCR (qRT-PCR)

RNA was extracted from Caco-2 cells using the RNArep Pure Cell/Bacteria Kit, while the reverse transcription and fluorescence quantification were carried out with FastKing one-step reverse transcription fluorescence quantification kits (probe method). Both kits were procured from TIANGEN Biotech (Beijing) Co., Ltd. in Beijing, China. Gene expression was assessed utilizing a Light Cycler 480 real-time PCR system from Roche, based in Basel, Switzerland. The amplification protocol consisted of an initial step at 95 °C for 30 s, followed by 45 cycles of 95 °C for 5 s and 60 °C for 30 s. Glyceraldehyde-3-phosphate dehydrogenase (GAPDH) served as the internal control gene and was analyzed using the 2^−ΔΔCt^ method. The sequences of the primers can be found in Table 1.

### 2.2. Broiler Experiment

#### 2.2.1. Ethics Statement

The research was licensed by the ethical approval of the Animal Care and Use Committee of the Institute of Feed Research of the Chinese Academy of Agricultural Science (Statement No. AEC-CAAS-20240315, Beijing, China).

#### 2.2.2. Experimental Design and Bird Management

A total of 240 one-day-old Arbor Acres (AA, 41.2 ± 0.5 g) broiler chickens were utilized in the experiment, which were randomly allocated into four treatment groups, each with six replicates containing ten broilers. The duration of the experiment was 21 d. The control group (CK) was provided with a basic diet (Table 2) designed to meet or surpass the standards outlined by NY-T 33-2004 (2004) [13]. Based on the CK group, the LPS group received an abdominal injection of 0.5 mg/kg body weight of LPS daily (19–21 d). In the JF group, the feed was supplemented with JF, building upon the CK group feed, resulting in a final concentration of JF in the JF group feed of 1 × 10^7^ CFU/g, achieved by gradually pre-mixing JF bacterial powder. Based on the JF group, broiler chickens were abdominally injected with 0.5 mg/kg of body weight of LPS. The JF+LPS group of broiler chickens received an abdominal injection of 0.5 mg/kg body weight of LPS daily (19–21 d) in addition to the treatment administered to the JF group. The feeding management of the AA broiler chickens adhered to the guidelines specified in the AA broiler chicken feeding management manual, which is consistent with our previous feeding protocols [13]. This management strategy was implemented at the Nankou Experimental Base of the Chinese Academy of Agricultural Sciences in Beijing, China.

#### 2.2.3. Sample Collection and Parameter Determination

On the 21st day, 2 healthy broiler chickens with similar weights were selected from each replicate, and their jejunums were separated. The antioxidant and cytokine indicators in the jejunal tissue of the broilers were assessed using a kit (Jiangsu Meimian Industrial Co., Ltd., Jiangsu, China). The analysis focused on measuring the levels of total antioxidant capacity (T-AOC), superoxide dismutase (SOD), catalase (CAT), malondialdehyde (MDA), glutathione peroxi dase (GSH-Px), tumor necrosis factor-alpha (TNF-α), interleukin-1 beta (IL-1β), interleukin-6 (IL-6), interleukin-8 (IL-8), interleukin-4 (IL-4), and interleukin-10 (IL-10) within the jejunal tissue.

On the 21st day, from each treatment group, three broiler chickens with similar body weights were chosen. The jejunum was subsequently isolated, and the expression of mRNA for jejunum-related genes was assessed. The methods used were in accordance with Section 2.1.7, and the sequences of the primers are shown in Table 3.

### 2.3. Statistical Analysis

The normality of distribution and the equality of variances in the data were evaluated using the Shapiro–Wilk and Levene’s tests. For data analysis, a one-way ANOVA was performed in conjunction with the General Linear Model (GLM) using SAS version 9.4 (SAS Institute Inc., Cary, NC, USA). Tukey’s multiple range tests were employed for comparisons between treatments. The results were reported as the mean with the standard error of the mean (SEM). Graphical representations of the experimental data were created with GraphPad Prism 8 software (GraphPad Software, San Diego, CA, USA). Statistically significant differences among the treatments are denoted by an asterisk (*) for *p* < 0.05 and by two asterisks (**) for *p* < 0.01.

## 3. Results

### 3.1. Cellular Antioxidant Capacity in Caco-2 Cells

**Figure 1:**
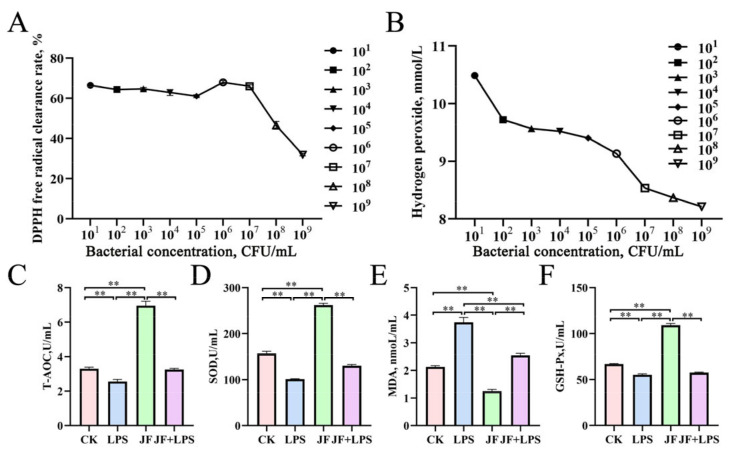
Cellular antioxidant capacity in Caco-2 cells. (**A**) DPPH free radical clearance rate; (**B**) hydrogen peroxide content; (**C**) T-AOC, total antioxidant capacity; (**D**) SOD, superoxide dismutase; (**E**) MDA, malondialdehyde; (**F**) GSH-Px, glutathione peroxidase. CK group, control check group; LPS group, lipopolysaccharide group; JF group, *Bacillus amyloliquefaciens* group; JF+LPS group, *Bacillus amyloliquefaciens* + lipopolysaccharide group. * There was a significant difference between the two treatment groups, where * represents *p* < 0.05, ** represents *p* < 0.01. Results are presented as the mean and standard error of the mean (SEM), n = 3.

As shown in Figure 1, the JF strain exhibited the highest DPPH free radical clearance rate (Figure 1A) at a concentration of 10^6^ CFU/mL (*p* < 0.05). Additionally, the hydrogen peroxide content (Figure 1B) demonstrated a significant decrease with increasing concentrations of the JF strain (*p* < 0.05). In comparison to the CK group, the JF group significantly enhanced the T-AOC content (Figure 1C), as well as SOD (Figure 1D) and GSH-Px (Figure 1F) activities (*p* < 0.05), while concurrently reducing the MDA (Figure 1E) content, in Caco-2 cells (*p* < 0.05). This finding contrasts with the LPS group, which showed a significant increase in MDA content and a decrease in T-AOC content, alongside reductions in SOD and GSH-Px activity (*p* < 0.05). Notably, the MDA content in the JF+LPS group was significantly lower than that observed in the LPS group (*p* < 0.05).

### 3.2. mRNA Expression of Cellular Antioxidant in Caco-2 Cells

**Figure 2:**
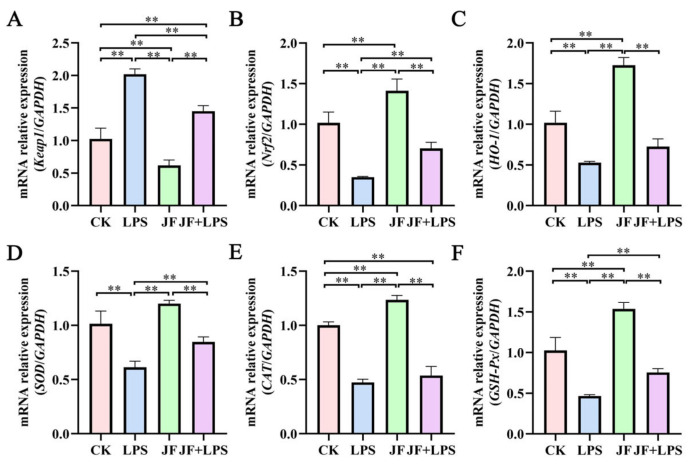
mRNA expression of cellular antioxidant in Caco-2 cells. (**A**) mRNA relative expression (*Keap1*/*GAPDH*); (**B**) mRNA relative expression (*Nrf2*/*GAPDH*); (**C**) mRNA relative expression (*HO-1*/*GAPDH*); (**D**) mRNA relative expression (*SOD*/*GAPDH*); (**E**) mRNA relative expression (*CAT*/*GAPDH*); (**F**) mRNA relative expression (*GSH-Px*/*GAPDH*). CK group, control check group; LPS group, lipopolysaccharide group; JF group, *Bacillus amyloliquefaciens* group; JF+LPS group, *Bacillus amyloliquefaciens* + lipopolysaccharide group. * There was a significant difference between the two treatment groups, where * represents *p* < 0.05, ** represents *p* < 0.01. Results are presented as the mean and standard error of the mean (SEM), n = 3.

As illustrated in Figure 2, the LPS group demonstrated a notable rise in the mRNA expression of *Keap1* (Figure 2A) within the Caco-2 cells (*p* < 0.05). At the same time, there was a reduction in the levels of mRNA expression for *Nrf2* (Figure 2B), *HO-1* (Figure 2C), *SOD* (Figure 2D), *CAT* (Figure 2E), and *GSH-Px* (Figure 2F) when evaluated against the CK group (*p* < 0.05). Conversely, the JF group led to a significant decrease in *Keap1* mRNA expression while increasing the levels of *Nrf2*, *HO-1*, *CAT,* and *GSH-Px* (*p* < 0.05). When compared to the LPS group, the JF+LPS group showed a significant decline in *Keap1* mRNA expression (*p* < 0.05), as well as substantial increases in the mRNA levels of *Nrf2*, *SOD*, and *GSH-Px* (*p* < 0.05).

### 3.3. mRNA Expression of Cellular Inflammatory Factors in Caco-2 Cells

**Figure 3:**
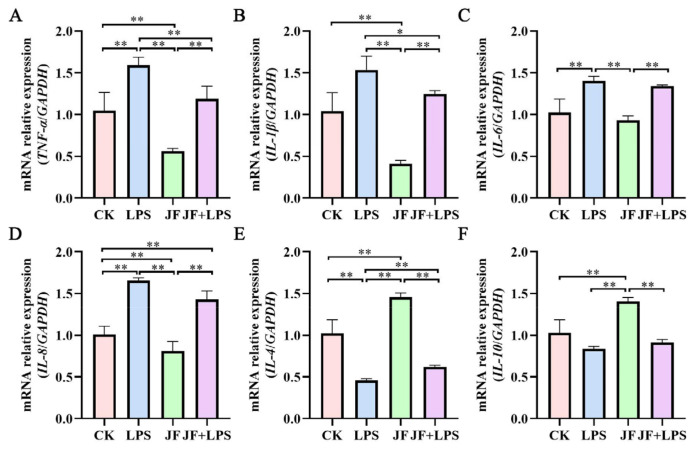
mRNA expression of cellular inflammatory factors in Caco-2 cells. (**A**) mRNA relative expression (*TNF-α*/*GAPDH*); (**B**) mRNA relative expression (*IL-1β*/*GAPDH*); (**C**) mRNA relative expression (*IL-6*/*GAPDH*); (**D**) mRNA relative expression (*IL-8*/*GAPDH*); (**E**) mRNA relative expression (*IL-4*/*GAPDH*); (**F**) mRNA relative expression (*IL-10*/*GAPDH*). CK group, control check group; LPS group, lipopolysaccharide group; JF group, *Bacillus amyloliquefaciens* group; JF+LPS group, *Bacillus amyloliquefaciens* + lipopolysaccharide group. * There was a significant difference between the two treatment groups, where * represents *p* < 0.05, ** represents *p* < 0.01. Results are presented as the mean and standard error of the mean (SEM), n = 3.

As illustrated in Figure 3, a substantial increase in the mRNA expression levels of *TNF-α* (Figure 3A), *IL-1β* (Figure 3B), *IL-6* (Figure 3C), and *IL-8* (Figure 3D) was observed in Caco-2 cells within the LPS group when compared to the CK group (*p* < 0.05). Simultaneously, a decrease in the mRNA expression level of *IL-4* (Figure 3E) was noted (*p* < 0.05). In contrast, the JF group showed a significant reduction in the mRNA expression levels of *TNF-α*, *IL-1β*, and *IL-8* (*p* < 0.05), while the mRNA expression levels of *IL-4* and *IL-10* were found to be elevated (Figure 3F) (*p* < 0.05). Importantly, the JF+LPS group demonstrated significantly lower mRNA expression levels of *TNF-α* and *IL-1β* compared to the LPS group (*p* < 0.05), with a marked increase in the mRNA level of *IL-4* (*p* < 0.05).

### 3.4. mRNA Expression of Cellular Intestinal Barrier Function in Caco-2 Cells

**Figure 4:**
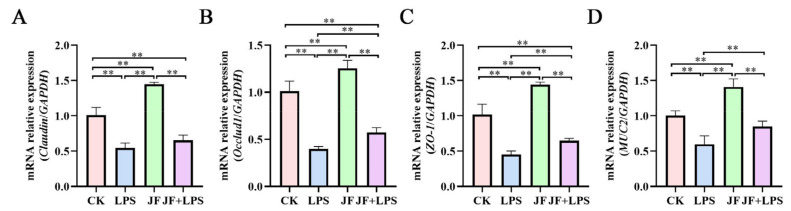
mRNA expression of cellular intestinal barrier function in Caco-2 cells. (**A**) mRNA relative expression (*Claudin*/*GAPDH*); (**B**) mRNA relative expression (*Occludin1*/*GAPDH*); (**C**) mRNA relative expression (*ZO-1*/*GAPDH*); (**D**) mRNA relative expression (*MUC2*/*GAPDH*). CK group, control check group; LPS group, lipopolysaccharide group; JF group, *Bacillus amyloliquefaciens* group; JF+LPS group, *Bacillus amyloliquefaciens* + lipopolysaccharide group. * There was a significant difference between the two treatment groups, where * represents *p* < 0.05, ** represents *p* < 0.01. Results are presented as the mean and standard error of the mean (SEM), n = 3.

As shown in Figure 4, the LPS group exhibited a significant reduction in the mRNA expression levels of *Claudin* (Figure 4A), *Occludin1* (Figure 4B), *ZO-1* (Figure 4C), and *MUC2* (Figure 4D) in the Caco-2 cells compared to the CK group (*p* < 0.05). Conversely, the JF group demonstrated a significant increase in the mRNA expression of *Claudin*, *Occludin*, *ZO-1*, and *MUC2* (*p* < 0.05). Furthermore, when compared to the LPS group, the JF+LPS group showed a significant increase in the mRNA expression levels of *Occludin*, *ZO-1*, and *MUC2* (*p* < 0.05).

### 3.5. Antioxidant Index of Broiler Jejunum

**Figure 5:**
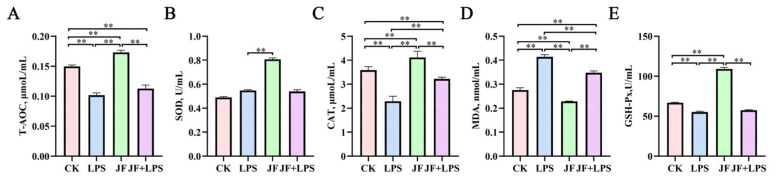
Antioxidant index of 21 d broiler jejunum. (**A**) T-AOC, total antioxidant capacity; (**B**) SOD, superoxide dismutase; (**C**) CAT, catalase; (**D**) MDA, malondialdehyde; (**E**) GSH-Px, glutathione peroxidase. CK group, control check group; LPS group, lipopolysaccharide group; JF group, *Bacillus amyloliquefaciens* group; JF+LPS group, *Bacillus amyloliquefaciens* + lipopolysaccharide group. * There was a significant difference between the two treatment groups, where * represents *p* < 0.05, ** represents *p* < 0.01. Results are presented as the mean and standard error of the mean (SEM), n = 6.

As shown in Figure 5, the LPS group exhibited a significant reduction in the activities of T-AOC (Figure 5A), CAT (Figure 5C), and GSH-Px (Figure 5E) in the jejunum of broiler chickens compared to the CK group (*p* < 0.05), alongside a notable increase in MDA (Figure 5D) content (*p* < 0.05). Conversely, the JF group demonstrated a significant enhancement in the activities of T-AOC, CAT, and GSH-Px (*p* < 0.05), while concurrently reducing MDA content (*p* < 0.05). When comparing the JF+LPS group to the LPS group, there was a significant increase in CAT content and a significant decrease in MDA content (*p* < 0.05).

### 3.6. Inflammatory Factors of Broiler Jejunum

**Figure 6:**
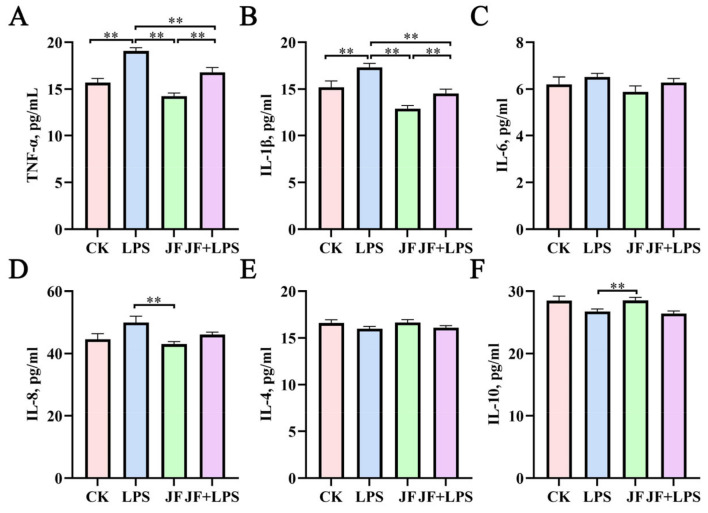
Inflammatory factors of 21 d broiler jejunum. (**A**) TNF-α, tumor necrosis factor-alpha; (**B**) IL-1β, interleukin-1β; (**C**) IL-6, interleukin-6; (**D**) IL-8, interleukin-8; (**E**) IL-4, interleukin-4; (**F**) IL-10, interleukin-10. CK group, control check group; LPS group, lipopolysaccharide group; JF group, *Bacillus amyloliquefaciens* group; JF+LPS group, *Bacillus amyloliquefaciens* + lipopolysaccharide group. * There was a significant difference between the two treatment groups, where * represents *p* < 0.05, ** represents *p* < 0.01. Results are presented as the mean and standard error of the mean (SEM), n = 6.

As shown in Figure 6, the levels of TNF-α (Figure 6A) and IL-1β (Figure 6B) in the jejunum of broiler chickens from the LPS group were significantly higher than those in the CK group (*p* < 0.05). However, when compared to the LPS group, the JF+LPS group exhibited a significant decrease in the levels of TNF-α and IL-1β (*p* < 0.05).

### 3.7. Expression of Antioxidant-Related mRNA in Broiler Jejunum

**Figure 7:**
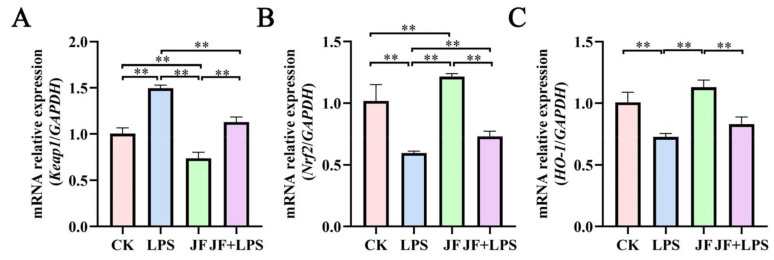
Expression of antioxidant-related mRNA in 21 d broiler jejunum. (**A**) mRNA relative expression (Keap1/GAPDH); (**B**) mRNA relative expression (Nrf2/GAPDH); (**C**) mRNA relative expression (HO-1/GAPDH). CK group, control check group; LPS group, lipopolysaccharide group; JF group, *Bacillus amyloliquefaciens* group; JF+LPS group, *Bacillus amyloliquefaciens* + lipopolysaccharide group. * There was a significant difference between the two treatment groups, where * represents *p* < 0.05, ** represents *p* < 0.01. Results are presented as the mean and standard error of the mean (SEM), n = 3.

As illustrated in Figure 7, a significant increase in the mRNA expression of *Keap1* (Figure 7A) was observed in the jejunum of broiler chickens belonging to the LPS group, as compared to the CK group (*p* < 0.05). In contrast, there was a notable decrease in the mRNA expression of *Nrf2* (Figure 7B) and *HO-1* (Figure 7C) in the same LPS group (*p* < 0.05). On the other hand, in the jejunum of the JF group, a significant reduction in *Keap1* mRNA expression was recorded (*p* < 0.05), while *Nrf2* and *HO-1* mRNA expressions were significantly elevated (*p* < 0.05). Additionally, when the JF+LPS group was compared to the LPS group, a substantial decrease in *Keap1* mRNA expression was noted in the jejunum of the JF+LPS group (*p* < 0.05), whereas *Nrf2* mRNA expression showed a significant increase (*p* < 0.05).

### 3.8. Expression of Inflammatory Factor-Related mRNA in Broiler Jejunum

**Figure 8:**
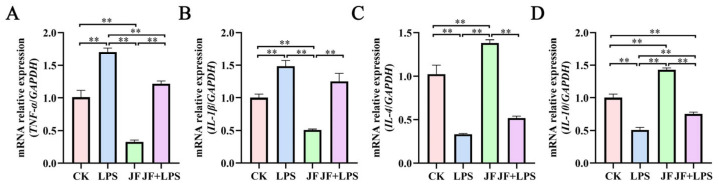
Expression of inflammatory factor-related mRNA in 21 d broiler jejunum. (**A**) mRNA relative expression (*TNF-α*/*GAPDH*); (**B**) mRNA relative expression (*IL-1β*/*GAPDH*); (**C**) mRNA relative expression (*IL-4*/*GAPDH*); (**D**) mRNA relative expression (*IL-10*/*GAPDH*). CK group, control check group; LPS group, lipopolysaccharide group; JF group, *Bacillus amyloliquefaciens* group; JF+LPS group, *Bacillus amyloliquefaciens* + lipopolysaccharide group. * There was a significant difference between the two treatment groups, where * represents *p* < 0.05, ** represents *p* < 0.01. Results are presented as the mean and standard error of the mean (SEM), n = 3.

As illustrated in Figure 8, the levels of mRNA expression for *TNF-α* (Figure 8A) and *IL-1β* (Figure 8B) in the jejunum of broiler chickens within the LPS group showed a significant increase when compared to the CK group (*p* < 0.05). Conversely, the JF group demonstrated a notable decrease in these mRNA levels (*p* < 0.05). In contrast, the JF group exhibited significantly elevated mRNA expression levels of *IL-4* (Figure 8C) and *IL-10* (Figure 8D) relative to the CK group (*p* < 0.05), while a significant reduction was observed in the LPS group (*p* < 0.05). Importantly, when comparing the JF+LPS group to the LPS group, there was a significant reduction in *TNF-α* mRNA expression (*p* < 0.05), but a notable increase in *IL-10* mRNA expression was recorded (*p* < 0.05).

### 3.9. Expression of Intestinal Barrier Function-Related mRNA in Broiler Jejunum

**Figure 9:**
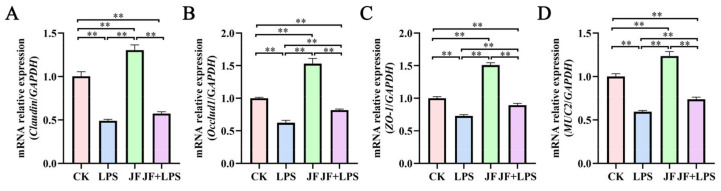
Expression of intestinal barrier function-related mRNA in 21 d broiler jejunum. (**A**) mRNA relative expression (*Claudin*/*GAPDH*); (**B**) mRNA relative expression (*Occludin1*/*GAPDH*); (**C**) mRNA relative expression (*ZO-1*/*GAPDH*); (**D**) mRNA relative expression (*MUC2*/*GAPDH*). CK group, control check group; LPS group, lipopolysaccharide group; JF group, *Bacillus amyloliquefaciens* group; JF+LPS group, *Bacillus amyloliquefaciens* + lipopolysaccharide group. * There was a significant difference between the two treatment groups, where * represents *p* < 0.05, ** represents *p* < 0.01. Results are presented as the mean and standard error of the mean (SEM), n = 3.

As illustrated in Figure 9, the mRNA levels of *Claudin* (Figure 9A), *Occludin1* (Figure 9B), *ZO-1* (Figure 9C), and *MUC2* (Figure 9D) in the jejunum of broiler chickens were significantly enhanced by JF when compared to the CK group (*p* < 0.05). In contrast, the LPS group exhibited a detrimental effect (*p* < 0.05). Additionally, in the JF+LPS group, there was a significant increase in the mRNA expression levels of *Occludin1*, *ZO-1*, and *MUC2* relative to the LPS group (*p* < 0.05).

## 4. Discussion

Broiler chickens are susceptible to oxidative stress under harmful stimuli from both internal and external environments [14]. This condition results in metabolic abnormalities and the rapid production of numerous highly reactive molecules, such as oxygen free radicals and nitric oxide [15]. The generation of these reactive oxygen species surpasses the reducing capacity of the body’s antioxidant system, leading to their accumulation within cells [16]. Consequently, this imbalance between oxidation and antioxidant systems precipitates oxidative damage processes. LPS is commonly employed to establish oxidative stress models [17]. It activates Toll-like receptor 4 (TLR4), triggering inflammatory responses that indirectly result in the generation of reactive oxygen species (ROS) [18]. This mechanism positions LPS as an ideal reagent for constructing oxidative stress models.

Probiotics are commonly utilized to enhance the body’s oxidative stress status. The antioxidant capacity of specific strains is typically assessed through the DPPH free radical clearance rate and hydrogen peroxide content [19]. Liu et al. demonstrated that selenium-rich *Bacillus amyloliquefaciens* C-1 could significantly reduce superoxide radicals, hydroxyl radicals, and DPPH levels in Caco-2 cells [20]. In this experiment, *Bacillus amyloliquefaciens* JF exhibited a higher DPPH free radical clearance rate, and the H_2_O_2_ content significantly decreased with increasing concentration. This result suggests that *Bacillus amyloliquefaciens* JF may play a significant role in antioxidant activity and cellular protection. Furthermore, the evaluation of the body’s oxidative stress status is generally conducted using five indicators: total antioxidant capacity (T-AOC), superoxide dismutase (SOD), catalase (CAT), glutathione peroxidase (GSH-Px), and malondialdehyde (MDA) [21,22]. Previous studies have demonstrated that the inclusion of 1.27 × 10^9^ CFU/g of *Bacillus amyloliquefaciens* CECT 5940 in the diet significantly enhances the serum activities of GSH-Px and SOD in broiler chickens, while simultaneously reducing MDA content [23]. Wang et al. demonstrated that the addition of 1 × 10^8^ CFU/g of *Bacillus amyloliquefaciens* SC06 significantly enhances the levels of T-AOC and T-SOD in the livers of broilers [24]. Consistent with the findings of previous studies, *Bacillus amyloliquefaciens* JF significantly enhanced the antioxidant capacity of the broiler jejunum. It increased the levels of T-AOC, CAT, and GSH-Px, while mitigating the decrease in CAT and the increase in MDA induced by LPS. The results indicate that Bacillus amyloliquefaciens JF can significantly enhance the antioxidant defense system in the intestines of broilers, thereby improving the body’s resistance to oxidative stress through the upregulation of key antioxidant enzymes.

The Keap1/Nrf2 signaling pathway is a crucial cellular signaling pathway that plays a significant role in maintaining cellular homeostasis and combating oxidative stress [25]. Its primary function is to regulate the antioxidant response of cells and mitigate oxidative stress damage by activating the expression of various antioxidant genes [26]. Among them, four antioxidant enzymes (HO-1, SOD, CAT, and GSH-PX), play crucial roles in the organism [27]. These enzymes collaborate to maintain redox balance and promote cellular health. Previous studies have demonstrated that *Bacillus amyloliquefaciens* SC06 alleviates oxidative stress in IPEC-J2 cells by modulating the Nrf2/Keap1 signaling pathway and reducing ROS production [28]. *Bacillus amyloliquefaciens* LSG2-8 significantly enhances the antioxidant capacity of Rhynchocypris lagowskii via the Keap1/Nrf2 signaling pathway [29]. Similarly, Wang et al. demonstrated that *Bacillus amyloliquefaciens* SC06 decreased the production of ROS and MDA in the jejunum of piglets by activating the Nrf2/Keap1 pathway, a finding that was corroborated in IPEC-J2 cells [30]. In this experiment, JF significantly enhanced the antioxidant capacity of Caco-2 cells and the broiler jejunum by modulating the Keap1/Nrf2 signaling pathway. Additionally, JF increased the mRNA expression of relevant antioxidant genes, including *HO-1*, *CAT*, and *GSH-Px*, while mitigating the effects of LPS that lead to the upregulation of *Keap1* and downregulation of *Nrf2*.

Under oxidative stress conditions, an excess of ROS can damage cellular proteins, lipids, and DNA, resulting in cellular injury [31]. These damaged cells can produce endogenous damage-related molecular patterns and release cytokines, which in turn recruit and activate additional inflammatory cells, thereby eliciting a systemic inflammatory response in the body [32]. The pro-inflammatory factors TNF-α, IL-1β, IL-6, and IL-8, along with the anti-inflammatory factors IL-4 and IL-10, play a crucial role in regulating the body’s inflammatory state [33]. Wang et al. found that *Bacillus amyloliquefaciens* SC06 significantly increased the intestinal content of IL-10, while concurrently reducing levels of TNF-α, in piglets [34]. Additionally, the team discovered that *Bacillus amyloliquefaciens* SC06 mitigated the production of intestinal inflammatory responses in piglets and inhibited intestinal cell apoptosis, thereby counteracting the detrimental effects induced by LPS [35]. In this experiment, *Bacillus amyloliquefaciens* JF significantly reduced the levels of LPS-induced pro-inflammatory cytokines TNF-α and IL-1β in the jejunum of broiler chickens. Furthermore, JF significantly decreased the mRNA expression levels of *TNF-α* and *IL-1β* in both Caco-2 cells and the jejunum of broiler chickens, while concurrently increasing the mRNA expression levels of *IL-4* and *IL-10*. This suggests that *Bacillus amyloliquefaciens* JF possesses the ability to regulate intestinal inflammatory responses, thereby contributing to the maintenance of intestinal immune homeostasis.

The intestinal barrier is a vital defense system of the body, essential for maintaining the stability and health of the internal environment [36]. Claudin, Occludin-1, ZO-1, and MUC2 are significant molecules associated with cell connectivity and mucosal barrier function [37]. These molecules play a critical role in preserving tissue structure and functional integrity and are regulated by a variety of influencing factors. Research has demonstrated that *Bacillus amyloliquefaciens* LSG2-8 enhances the intestinal barrier function of Macrobrachium rosenbergii by elevating the mRNA expression levels of *ZO-1* and *claudin-3* [38]. Du et al. demonstrated that, in piglets, *Bacillus amyloliquefaciens* SC06 enhances the barrier function of intestinal epithelial cells by improving the structure of the intestinal mucosa and the expression of tight junction proteins [39]. Consistent with previous research findings, *Bacillus amyloliquefaciens* JF significantly enhances the mRNA expression of *Claudin*, *Occludin-1*, and *MUC2* in Caco-2 cells and the jejunum of broilers, while also markedly improving the intestinal barrier damage induced by LPS. JF can significantly enhance the integrity of tight junctions between intestinal epithelial cells and promote the formation of the mucus layer in broiler chickens, thereby preventing harmful substances, such as LPS, from penetrating the intestinal wall and entering the body. This mechanism serves to protect intestinal health.

## 5. Conclusions

In summary, *Bacillus amyloliquefaciens* JF improves the oxidative stress status of Caco-2 cells and the jejunum of broilers by modulating the Keap1/Nrf2 signaling pathway. Additionally, it reduces intestinal inflammation levels and improves intestinal barrier function.

## Figures and Tables

**Table 1:** Real time-PCR primers of Caco-2 cells.

Target (Human)	Primer Sequence (5′-3′)	Accession NO.
GAPDH	F:GACCCCTTCATTGACCTCAAC R:CATACCAGGAAATGAGCTTG	NM_002046.7
Keap1	F:CCTTCAGCTACACCCTGGAG R:AACATGGCCTTGAAGACAGG	NM_016679.4
Nrf2	F:AGACAAACATTCAAGCCGCT R:CCATCTCTTGTTTGCTGCAG	NM_010902.4
HO-1	F:CAGTCTTCGCCCCTGTCTAC R:GCTGGTGTGTAGGGGATGAC	NM_002133.2
SOD	F:GAAGGTGTGGGGAAGCATTA R:ACCACAAGCCAAACGACTTC	NM_000454
CAT	F:ACATGGTCTGGGACTTCTGG R:CTTGGGTCGAAGGCTATCTG	NM_001752.3
GSH-Px	F:AACCAGTTTGGGCATCAGG R:GTTCACCTCGCACTTCTCG	NM_001329455.1
TNF-α	F:TGTAGCCCATGTTGTAGCAAACC R:GAGGACCTGGGAGTAGATGAGGTA	NM_000594.3
IL-1β	F: AAGCTGATGGCCCTAAACAG R:AGGTGCATCGTGCACATAAG	NM_000576.2
IL-4	F:TCTTTGCTGCCTCCAAGAACA R:GTAGAACTGCCGGAGCACAG	NM_000589.4
IL-6	F:TGGCTGAAAAAGATGGATGCT R:TCTGCACAGCTCTGGCTTGT	NM_000600.5
IL-8	F:CTGGCCGTGGCTCTCTTG R:GGGTGGAAAGGTTTGGAGTATG	NM_000584.3
IL-10	F:TCTCCGAGATGCCTTCAGCAGA R:TCAGACAAGGCTTGGCAACCCA	NM_000572.2
Claudin	F:AGTTAGGAGCCTTGATGCCG R:GCACAGGGAGTAGGATACGC	NM_021101.5
Occludin-1	F:CTTCCAATGGCAAAGTGAATGAATGAC R:TACCACCGCTGCTGTAACGAG	NM_002538
ZO-1	F:GAGCCTAATCTGACCTATGAACC R:TGAGGACTCGTATCTGTATGTGG	NM_003257.3
MUC2	F:CTTATCTGCTGTGTCCTGAA R:AAGTCTCGTTCTCCTGTCT	NM_002457.5

Keap1, Kelch-like ECH-associated protein 1; Nrf2, nuclear factor-erythroid 2-related factor 2; HO-1, heme oxygenase-1; SOD, superoxide dismutase; CAT, catalase; GSH-Px, glutathione peroxidase; TNF-α, tumor necrosis factor-alpha; IL-1β, interleukin-1β; IL-4, interleukin-4; IL-6, interleukin-6; IL-8, interleukin-8; IL-10, interleukin-10; ZO-1, zonula occludens-1; MUC2, mucin 2.

**Table 2:** Ingredients and nutrient content of basal diet (%, as-is basis).

Items	Contents
	Starter (1–21 d)
Corn	56.593
Soybean meal	29.083
Cottonseed meal	3.024
Plant oil	1.794
DDGS	5.040
CaHPO_4_	1.583
NaCl	0.313
Limestone	1.532
*DL*-methionine	0.272
*L*-lysine	0.413
Choline chloride	0.202
Threonine	0.020
Premix ^1^	0.131
Total	100.000
Nutrient content	
Metabolisable energy, MJ/kg	12.55
Crude protein	20.00
Calcium, %	1.02
Available phosphorus	0.45
Phosphorus	0.62
Lysine, %	1.25
Methionine, %	0.58
Methionine + cysteine, %	0.90
Threonine, %	0.80
Tryptophan, %	0.25

^1^ The premix provided the following per kg of diets: VA 8 000 IU, VD_3_ 1 000 IU, VE 20 IU, VK_3_ 0.5 mg, VB_1_ 2 mg, VB_2_ 8 mg, VB_3_ 10 mg, VB_5_ 40 mg, VB_6_ 3.5 mg, VB_11_ 0.3 mg, VB_12_ 0.01 mg, biotin 0.12 mg, Cu (as copper sulfate) 8 mg, Fe (as ferrous sulfate) 80 mg, Mn (as manganese sulfate) 60 mg, Zn (as zinc sulfate) 40 mg, Se (as sodium selenite) 0.15 mg, I (as potassium iodide) 0.7 mg.

**Table 3:** Real-time PCR primers of broiler chickens.

Target (Broiler)	Primer Sequence (5′-3′)	Accession NO.
GAPDH	F:TGTTGCCATCAATGACCCCTTT R:CTCCACGACGTACTCAGCG	NM_204305.2
Keap1	F:CAGCGTGAGAGGTGAGTATGAG R:TACAGCAGTCGGTTCAGCACT	XM_010728179.2
Nrf2	F:TCGCAGAGCACAGATACTTCAA R:CTGGAGAAGCCTCATTGTCATCTA	NM_001396902.1
HO-1	F:GTCCCGAATGAATGCCCTTGA R:ATGACCGTTCTCCTGGCTCTT	NM_205344.2
TNF-α	F:CCCCTACCCTGTCCCACAA R:TGAGTACTGCGGAGGGTTCAT	NM_204267
IL-1β	F:ACTGGGCATCAAGGGCTA R:GGTAGAAGATGAAGCGGGTC	NM_204524.1
IL-4	F:ACATCCAGGGAGAGGTTTCCT R:CGTGTTGAGGAAGAGACCCTG	XM_046900385.1
IL-10	F:TCTGTGTCAGAGATGCTGCG R:CAGGTGAAGAAGCGGTGACAG	NM_214041.1
Claudin	F:ACATTGGTTCAAGCATCGTGAC R:GCTGTAGATGTCGCACTGAGT	NM_001013611
Occludin1	F:ATCAACGACCGCCTCAATCAG R:TCCTCTGCCACATCCTGGTATT	NM_205128.1
ZO-1	F:CCACCTCAGAATAAGCCAGCAAT R:CGGTTGTAAGAAGGAGTGACTGTT	XM_413773.4
MUC2	F:TTCATGATGCCTGCTCTTGTG R:CCTGAGCCTTGGTACATTCTTGT	JX284122.1

## Data Availability

The original contributions presented in this study are included in the article; further inquiries can be directed to the corresponding author.
